# Hydrogen Peroxide Mediates Artemisinin-Derived C-16 Carba-Dimer-Induced Toxicity of Human Cancer Cells

**DOI:** 10.3390/antiox9020108

**Published:** 2020-01-26

**Authors:** Amanda L. Kalen, Brett A. Wagner, Ehab H. Sarsour, Maneesh G. Kumar, Jessica L. Reedy, Garry R. Buettner, Nabin C. Barua, Prabhat C. Goswami

**Affiliations:** 1 Free Radical and Radiation Biology Division, Department of Radiation Oncology, The University of Iowa, Iowa City, IA 52242, USA; amanda-kalen@uiowa.edu (A.L.K.); brett-wagner@uiowa.edu (B.A.W.); mgkumar@gmail.com (M.G.K.); garry-buettner@uiowa.edu (G.R.B.); 2 Department of Basic Sciences, Kansas City University of Medicine and Biosciences, Kansas City, MO 64106, USA; esarsour@kcumb.edu; 3 Radiation Oncology Branch, Center for Cancer Research, National Cancer Institutes of Health, Bethesda, MD 20892, USA; jess.reedy@nih.gov; 4 Natural Products Chemistry Division, CSIR-North East Institute of Science and Technology, Jorhat, 785006 Assam, India; ncbarua2000@yahoo.co.in

**Keywords:** artemisinin, carba-dimer, aliphatic nitro chemistry, oxidative stress, cyclin D1

## Abstract

This study used a nitroaliphatic chemistry approach to synthesize a novel artemisinin-derived carba-dimer (AG-1) and determined its anti-proliferative effects in human normal and cancer cells. AG-1 treatments selectively inhibit proliferation of cancer cells compared to normal human fibroblasts. Compared to artemisinin, AG-1 is more toxic to human breast, prostate, head–neck, pancreas and skin cancer cells; 50% inhibition (IC_50_) 123 µM in AG-1 vs. 290 µM in artemisinin-treated breast cancer cells. AG-1 treatment decreased (~5 folds) cyclin D1 protein expression that correlated with an increase in the percentage of cells in the G_1_-phase, suggesting a G_1_ delay. AG-1-induced toxicity was independent of the DNA damage at 72 h post-treatment, as measured by micronuclei frequency and γH2AX protein levels. Results from electron paramagnetic resonance spectroscopy showed Fe-catalyzed formation of AG-1 carbon-centered radicals in a cell-free system. Flow cytometry analysis of H_2_DCF-DA oxidation showed a significant increase in the steady-state levels of reactive oxygen species (ROS) in AG-1-treated cells. Pre-treatment with *N*-acetyl-l-cysteine and antioxidant enzymes (superoxide dismutase and catalase) significantly suppressed AG-1-induced toxicity, suggesting that superoxide and hydrogen peroxide contribute to AG-1-induced toxicity in human cancer cells. AG-1 represents a novel class of anti-cancer drug that is more potent than its parent compound, artemisinin.

## 1. Introduction

Natural products have a long history of use in the treatment of numerous human diseases. In addition to their useful biological activities, many of these chemicals are used as lead compounds in the chemical synthesis of the next generation of pharmaceuticals designed to improve therapy outcome. Artemisinin (also known as qinghaosu or sweet wormwood), 1,2,4-trioxane sesquiterpene, is an active ingredient of the *Artemisia annua* plant commonly found in Asia. Historically, this plant has been used by ancient Chinese herbalists to treat high fever. The active ingredient, artemisinin was first isolated in 1972 by Youyou Tu [1]. Because of its high potency and low toxicity to normal cells, artemisinin has been approved by the Food and Drug Administration for the clinical management of malaria. Furthermore, ester and ether derivatives of artemisinin (lactol, artemether, arteether, and artesunate) are currently being examined to treat multi-drug (quinine-, chloroquine-, and mefloquine-) resistant strains of malaria parasites [2].

In addition to its well-known anti-malarial effects, recent evidence also suggests that artemisinin and its derivatives have anti-cancer properties [3,4,5,6]. Oral administration of artemisinin has been shown to inhibit 7,12-dimethylbenz(a)anthracene induced carcinogenesis in a rat model of mammary cancer [3]. The Developmental Therapeutics Program of the National Cancer Institute, USA, analyzed the ester-derivative of artemisinin-monomer (artesunate) in 55 cancer cell lines and showed that artesunate has anti-cancer properties in cell lines representative of leukemia, melanoma, central nervous system, colon, prostate, ovarian, renal, and breast cancer [7]. Dihydroartemisinin has shown a potent anti-proliferative effect in leukemia, lung and ovarian cancers, and artemisone showed a similar effect in melanoma, breast, colon and pancreatic cancers [8,9]. Whereas the use of artemisinin and its derivatives as potential cancer therapy agents is gaining interest, the mechanisms regulating their anti-proliferative effects are not completely understood. It is believed that in the presence of iron, the endoperoxide (–C–O–O–C–) bridge in artemisinin can undergo redox-modification to generate carbon- and oxygen-centered radicals [2,10]. An additional pathway of free radical formation could be due to the generation of superoxide (or peroxyl radical) and an epoxide of artemisinin. Both superoxide and epoxide are anticipated to cause oxidative stress resulting in damage to cellular macromolecules and, subsequently, parasite death. It is currently unknown whether the same mechanisms of free radical generation regulate artemisinin-induced cytostatic and cytotoxic effects in cancer cells. 

A major limitation of the first-generation artemisinin derivatives (lactol, artemether, arteether, and artesunate) is the metabolic susceptibility of the C-10 acetal linkage, which undergoes rapid hydrolysis and is, subsequently, cleared by glucuronidation. The present study used a nitroaliphatic chemistry approach to synthesize an artemisinin-derived carba-dimer, (AG-1) with two endoperoxide (–C–O–O–C–) bridges. Results from an in vitro cell culture study show that compared to artemisinin, AG-1 is more effective in inducing oxidative stress and toxicity in human cancer cells. Pre-treatment with *N*-acetyl-l-cysteine and antioxidant enzymes (superoxide dismutase and catalase) suppressed AG-1-induced toxicity in cancer cells, suggesting that reactive oxygen species (ROS: superoxide and hydrogen peroxide) contribute to the toxicity of AG-1. 

## 2. Materials and Methods 

### 2.1. Analytical Chemistry Approach

Merck silica gel (100–200 mesh) column chromatography Sigma Aldrich, St. Louis, MO, USA and preparative thin-layer chromatography Sigma Aldrich, St. Louis, MO, USA were used to purify synthesized compounds. Compounds were identified and characterized using ^1^H NMR and ^13^C NMR (Bruker DPX-300, Bruker, Manning Park Billerica, MA, USA), IR (Perkin-Elmer 1640 FT-IR, PerkinElmer, Waltham, MA, USA) and Mass (WATERS Micro-mass ZQ 4000; ESI Probe, Waters, Milford, MA, USA) spectrometers. A Perkin-Elmer 343 Polari meter (PerkinElmer, Waltham, MA, USA) was used to measure optical rotations. A Buchi B-540 melting point apparatus (Sigma Aldrich, St. Louis, MO, USA) was used to record melting points. Reactions were conducted under dry nitrogen atmospheric conditions. Solvents were distilled prior to their use. 

### 2.2. Cell Culture and Reagents

The human breast (MDA-MB-231), melanoma (A375 and MD-435), prostate (PC-3), head–neck (Cal27) and pancreas (MIA PaCa-2) cancer cell lines were obtained from the American Type Culture Collection (ATCC, Manassas, VA, USA) and cultured in RPMI 1640 medium (GIBCO, Life Technologies, Gaithersburg, MD, USA) supplemented with 10% fetal bovine serum (FBS; HyClone, Salt Lake City, UT, USA) and antibiotics (penicillin and streptomycin) in a 37 °C humidified incubator with 5% CO_2_ and 95% air. Normal human fibroblasts were obtained from the Coriell Institute for Medical Research, Camden, NJ, USA. Stock solutions of artemisinin (Art) or its dimer (AG-1) were prepared in dimethyl sulfoxide and appropriate aliquots were added to monolayer cultures of normal and cancer cells. Cell numbers were counted using a Z1 Coulter Counter (Beckman Coulter, Indianapolis, IN, USA) at 2 day intervals. Cell population doubling time (T*_d_*) was calculated: T*_d_* = 0.693*t*/ln (N*_t_*/N*_0_*). Linear regression analysis was used to calculate the concentrations of artemisinin and AG-1 that resulted in 50% inhibition (IC_50_) in cell proliferation. Antibodies to human cyclin D1, phosphorylated H2AX (γH2AX) and actin were purchased from Upstate Technology (Otego, NY, USA) and Millipore Lifesciences (Burlington, MA, USA), respectively. Polyethylene glycol (PEG) conjugated catalase and superoxide dismutase were purchased from Sigma-Aldrich (St. Louis, MO, USA).

### 2.3. Toxicity Assay

Survival of cells was measured using a clonogenic assay. Control, artemisinin, and AG-1-treated cells were trypsinized and single-cell suspensions were plated in 60 mm tissue culture dishes. Cells were cultured for 14 d, fixed and stained with 0.005% crystal violet. Colonies containing 50 or more cells were counted. Surviving fraction (SF) was calculated after correction for plating efficiency (PE): PE = (number of colonies counted/number of cells seeded) × 100; SF = (number of colonies counted/number of cells seeded) × PE. Normalized SF was calculated relative to SF in untreated control cells. *N*-acetyl-l-cysteine (NAC, 5 mM) or polyethylene glycol (PEG) conjugated catalase (500 units mL^−1^ media; Sigma Aldrich, St. Louis, MO, USA) and superoxide dismutase (50 units mL^−1^ media; Sigma Aldrich, ST. Louis, MO, USA) were added to cell culture 3 h prior to the Art or AG-1 treatment. Toxicity was measured as described above. 

### 2.4. Flow Cytometry Assays

#### 2.4.1. Cell Cycle Phase Analysis

Flow cytometry analysis of DNA content was used to measure distributions of cell cycle phases following our published method [11]. Control and drug-treated monolayer cultures were trypsinized and fixed in 70% ethanol. Cells were treated with RNase A (0.1 mg mL^−1^) and incubated with 35 μg mL^−1^ of propidium iodide (PI). FACScan (Becton Dickinson, Franklin Lakes, NJ, USA), CellQuest Pro (Immunocytometry Systems, San Jose, CA, USA) and MODFIT software (Verity Software House, Topsham, ME, USA) were used to measure distributions of cells in different cell cycle phases. 

#### 2.4.2. Reactive Oxygen Species

Control and 15 min drug-treated cells were rinsed and incubated with Hanks buffer salt solution (HBSS) containing 1 µg mL^−1^ of 5-(and-6)-carboxy-2′,7′-dichlorodihydrofluorescein diacetate (H_2_DCF-DA, Invitrogen, Thermo Scientific, Waltham, MA, USA) for 15 min at 37 °C. Trypsinized cells were re-suspended in HBSS buffer supplemented with 10% FBS. H_2_DCF-oxidation, i.e., formation of DCF, was measured using 488 nm excitation and 530/30 nm band pass emission filter. Twenty thousand events were collected in the list mode and mean fluorescence intensity (MFI) was calculated using the Cell Quest software (Immunocytometry Systems, San Jose, CA, USA). Relative MFI was calculated with reference to the MFI of untreated control cells. 

#### 2.4.3. Glucose Uptake

Glucose uptake assay was performed following our previously published method) [12]. Control and drug-treated cells were rinsed and incubated with DMEM containing glucose (1 g L^−1^) and 2-(*N*-(7-nitrobenz-2-oxa-1,3-diazol-4-yl)amino)-2-deoxyglucose (2-NBDG; 20 µM) for 1 h. BD LSRII flow cytometer (Franklin Lakes, NJ, USA)) was used to measure fluorescence of 2-NBDG uptake; excitation at 465 nm, emission at 540 nm. Twenty thousand events were collected in the list mode and the mean fluorescence intensity (MFI) was calculated for each sample after correction for autofluorescence. Fold change in MFI was calculated relative to untreated control cells.

### 2.5. Electron Paramagnetic Resonance (EPR) Spectroscopy

The University of Iowa Holden Comprehensive Cancer Center EPR core facility was used to perform spin trapping experiments to determine whether AG-1 can form free radicals in a cell-free system. In this method, a diamagnetic compound (spin trap) reacts with short-lived paramagnetic free radicals to form spin adducts, which are detectable by EPR. The spin adducts have a longer half-life compared to the original free radical, allowing them to accumulate to a detectable level. The EPR spectrum of a spin-adduct is unique for the given radical (or type of radical), having characteristic hyperfine splitting constants [13]. AG-1 (1 mM) was incubated in PBS (pH 7.4) containing 25 mM of the spin trap α-(4-pyridyl-1-oxide)-*N*-*tert*-butylnitrone (POBN) in the presence of 20 µM or 100 µM ferrous sulfate. EPR spectra were recorded using a Bruker EMX spectrometer (Manning Park Billerica, MA, USA) with a magnetic field modulation frequency of 100 kHz and nominal microwave power 20 mW in a TM cavity and flat cell. The scans were collected with a modulation amplitude of 1 G, a scan rate of 200 G/42 s, a time constant of 41 ms and a receiver gain of 2.5 × 10^5^; ten scans were accumulated to generate spectra. Hyperfine splitting parameters were determined with the aid of spectral simulation using the National Institute of Environmental Health Science’s WinSIM EPR spectra simulation program [14].

### 2.6. DNA Damage Assay

Micronucleus assay was used to measure DNA damage following our published protocol [15]. Control, artemisinin and AG-1-treated monolayer cultures were rinsed and incubated with regular medium containing cytochalasin-B (4 μg mL^−1^). Trypsinized cells were fixed in Carnoy-fixative solution (methanol:acetic acid, 3:1) and spread onto microscopy slides. Cells were stained with 0.0125% acridine orange in Sorensen’s buffer (pH 6.8) and visualized under a fluorescent microscope; excitation wavelength, 453 nm and a 450–490 nm band pass filter (Olympus BX-51, Center Valley, PA, USA). A minimum of 200 binucleated cells with well-preserved cytoplasm were scored for each treatment. The percentage of micronucleated binucleate cells was calculated as described previously [15].

### 2.7. Immunoblotting Assay

Cell pellets were re-suspended in phosphate buffer (pH 7.4) containing PhosSTOP, (Sigma Aldrich, St. Louis, MO, USA) and a protease inhibitor cocktail (Sigma Aldrich, St. Louis, MO, USA). Cell suspensions were sonicated and the concentration of total proteins in each sample was measured using Bradford assay. Proteins were separated by 12% SDS-PAGE (Sodium dodecyl sulfate polyacrylamide gel electrophoresis) and transferred onto a nitrocellulose membrane using a semidry-electrophoresis transfer method [15]. The membrane was then incubated with antibodies to cyclin D1 (1:500) or γH2AX (1:200); amount of actin protein in each sample was used for loading correction. An enhanced chemiluminescence kit (Thermo Fisher Scientific, Madison, WI, USA) was used to visualize the immunoreactive polypeptide bands. NIH-ImageJ software was used to quantify results. γH2AX levels in 6 Gy (cesium^137^ source; 0.84 Gy min^−1^) irradiated cells were used as a positive control. 

### 2.8. Statistical Analysis

All results were compared with that of specific controls using the student *t*-test and ANOVA with posthoc test using GraphPad Prism version 4 software (San Diego, CA, USA). Results with *p* < 0.05 were considered significant. 

## 3. Results

### 3.1. Synthesis of AG1

**Figure 1:**
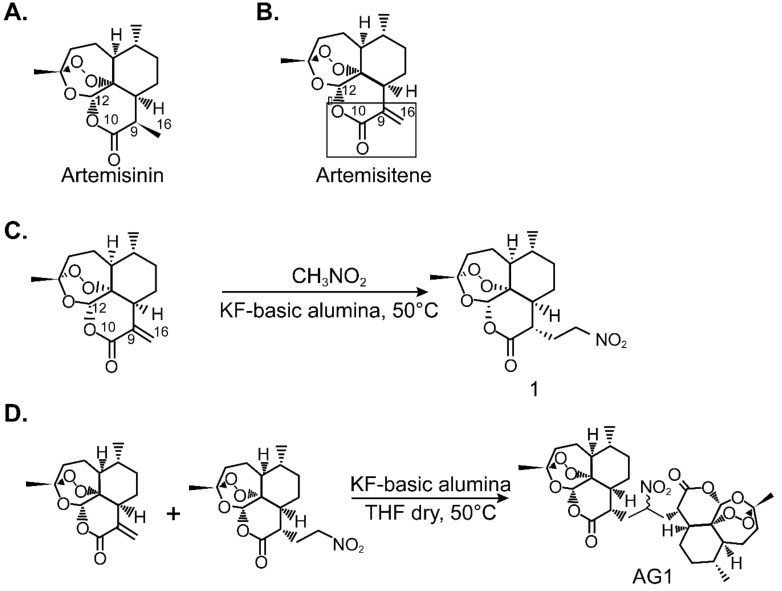
Synthesis of artemisinin-derived C-16 carba-dimer, AG-1. Nitroaliphatic chemistry was used to synthesize AG-1. (**A**) Artemisinin; (**B**) Artemisitene; (**C**) Scheme-1 for the synthesis of artemisinin-derived Michael adduct; **(D)** Scheme-2 for the artemisinin-derived C-16 carba-dimer, AG-1.

Nitroaliphatic chemistry [16], and artemisinin (Figure 1) were used to synthesize the C16 carba-dimer, AG-1. Artemisitene was synthesized from artemisinin (Figure 1A) by using a selenoxide elimination route [9]. A α-methylene lactone (Figure 1B) moiety is susceptible to undergo 1, 4 addition reaction to generate the corresponding Michael adduct. 

#### 3.1.1. Synthesis of Artemisinin-Derived Michael Adduct 

KF-basic alumina (0.1 g) was added to artemisitene (0.200 g, 0.712 mmol) dissolved in nitromethane and stirred at 50 °C for 2 h. Completion of the reaction was verified by thin-layer chromatography. Reaction mixture was filtered and concentrated. Column chromatography was used to isolate the nitro adduct (80% yield) and purified product was characterized (Figure 1C).

White solid, m.p. 114.4 °C, [α] _D_^20^ (c 1.7, CHCl_3_) = +57

^1^H NMR (300 MHz, CDCl_3_) δ 5.98 (s,1H), 4.87–4.71 (m, 1H), 4.67–4.59 (m, 1H), 2.69–2.64 (m, 1H), 2.46–1.73 (m, 13H),1.45 (s, 3H), 1.05 (d, 3H, J = 6Hz).^13^C NMR (75 MHz, CDCl_3_) δ 175.59, 110.45, 99.0, 84.96, 55.24, 49.43, 46.6, 42.44, 38.79, 36.82, 35.94, 30.34, 29.54, 25.1, 19.7, IR (CHCl3) 1725, 1547 cm^−1^, ESIMS m/e 341 (M+).

#### 3.1.2. Synthesis of Artemisinin Dimer, AG-1 

To a stirred solution of artemisitene (0.114 g, 0.205 mmol) in dry tetrahydrofuran (THF), 0.07 g, 0.205 mmol of the nitro adduct 1 was added and heated to 50 °C on an oil bath. KF-basic alumina (0.12 g) was added. After completion of the reaction, C-16 carba-dimer (AG-1) was purified by using preparative thin-layer chromatography (0.079g; 62% yield) and characterized (Figure 1D).

White crystals, m.p. 126–130 °C, [α]_D_^20^ (c 0.8, CHCl_3_) = +70.3

^1^H NMR (300 MHz, CDCl_3_) δ 5.94 (s, 1H), 5.92 (s, 1H), 5.30 (m, 1H), 2.72-1.6 (m, 28H), 1.46 (s, 3H), 1.44(s, 3H), 1.01 (d, 3H, J = 2.58 Hz), 0.99 (d, 3H, J = 2.4Hz).^13^C NMR (75 MHz, CDCl_3_) δ 171.21, 170.49, 133, 105.52, 94.19, 93.88, 84.93, 80.52, 80.09, 50.11, 45.69, 41.8, 41.53, 41.39, 39.88, 37.54, 35.84, 33.79, 30.93, 30.7, 25.45, 24.76, 24.65, 19.82. IR (CHCl_3_) 1736, 1548 cm^−1^, ESIMS m/e 622 (M+)

NMR spectroscopy was used to identify the stereochemical assignment at the C-9 carbon center of the artemisinin molecule. Ten shifting of C-9 α- proton at δ 3.39 of artemisinin to δ 2.69 in the newly generated C-16 artemisinin derivative clearly suggests α-stereochemistry at C-9 position. The nitro derivative of artemisinin was used in the synthesis of novel C-16 carba-dimer. This newly generated nucleophile underwent 1,4-conjugate addition with the artemisitene generating C-16 carba-dimer 2 (AG-1) with 62% yield.

### 3.2. AG-1 is More Cytotoxic Than Artemisinin (Art)

**Figure 2:**
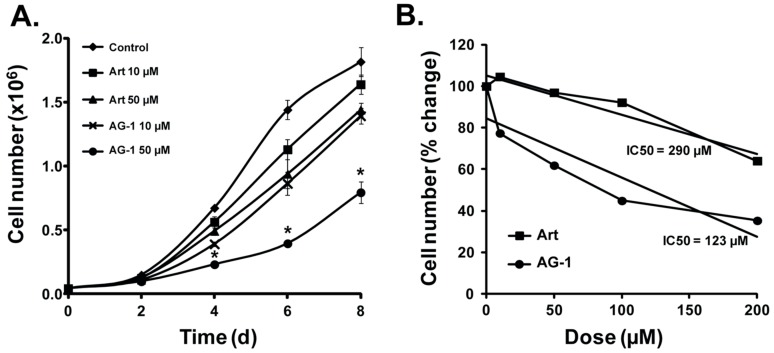
Artemisinin dimer (AG-1) treatment inhibits proliferation of MB-231 human mammary epithelial cancer cells. (**A**) Artemisinin (Art) dimer (AG-1) is more cytotoxic compared to Art. Twenty-four hours of asynchronous cultures of MB-231 were treated with 10 and 50 µM of Art or AG-1. Cells were continued in culture in the presence of the drug and cell numbers were counted at the time of addition of the drug (0 day) and then at 2 day intervals. Results are shown as average and standard deviation. Asterisks represent significance compared to untreated control cells; *n* = 3, *p* < 0.05. (**B**) A significant decrease in IC_50_ of AG-1 compared to Art-treated MB-231 cells. Cells were treated with 10-200 µM of Art or AG-1 and cell-number was counted at 72 hours after addition of the drug. Linear regression plot was used to calculate dose that resulted in 50% or more inhibition in cellular proliferation (IC_50_).

Tumor-derived cell lines are important tools for screening and identification of medicinal agents that can expedite the preclinical assessment of new anticancer drugs. To determine whether AG-1 is more potent compared to Art, MB-231 cells were incubated with 10 and 50 µM of Art and AG-1. Cell numbers were determined at the time of addition of the drugs and then at 2 day intervals (Figure 2A). Results showed a dose-dependent increase in MB-231 cell population doubling time in AG-1 compared to Art-treated cells. 

While untreated control cells exhibited a cell doubling time of 31 h, cells treated with10 µM AG-1 had a doubling time of 36 h compared to 32 h in 10 µM Art-treated cells. Cell doubling time increased to 45 h in 50 µM AG-1-treated cells compared to 34 h in 50 µM Art-treated cells. The observation that AG-1 is more efficient in inhibiting proliferation of MB-231 cells is also supported by its IC_50_ value (Figure 2B). Cells were treated with 0–200 µM Art and AG-1 for 72 h and cell number counted. Linear regression plot was used to calculate IC_50_. AG-1 was found to have a significantly lower IC_50_ value compared to Art: 123 vs. 290 µM. 

**Figure 3:**
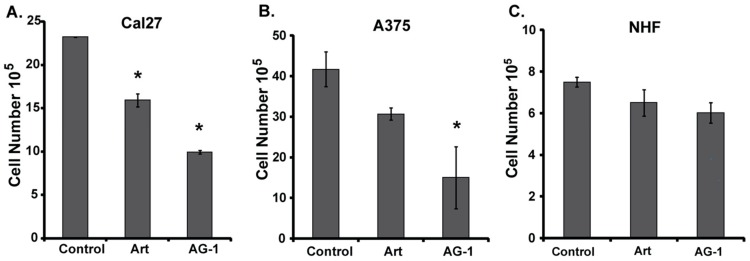
Artemisinin dimer (AG-1) treatments differentially inhibit proliferation of cancer vs. normal cells. Monolayer cultures of (**A**) Cal27 human head–neck cancer cells, (**B**) A375 human melanoma cells and (**C**) normal human fibroblasts (NHFs) were treated with 100 µM of Art and AG-1. Cell numbers were counted at 48–96 hours post-treatment. Asterisks represent significance compared to cell numbers in untreated control cells, *n* = 3, *p* < 0.05.

To determine whether AG-1 induces a differential growth delay in normal compared to cancer cells, monolayer cultures of normal human fibroblasts (NHFs) and cancer cells (Cal27 and A375) were treated with Art and AG-1 for 48–96 h. Cell numbers were counted using a Coulter Counter. Results showed both Art and AG-1 treatments significantly inhibited proliferation of Cal27 (Figure 3A) and A375 (Figure 3B) cancer cells whereas the same treatments did not affect proliferation of NHFs (Figure 3C). Overall, these results (Figure 2 and Figure 3) showed that AG-1 is more toxic compared to Art, and that both compounds were non-toxic to normal cells. 

**Figure 4:**
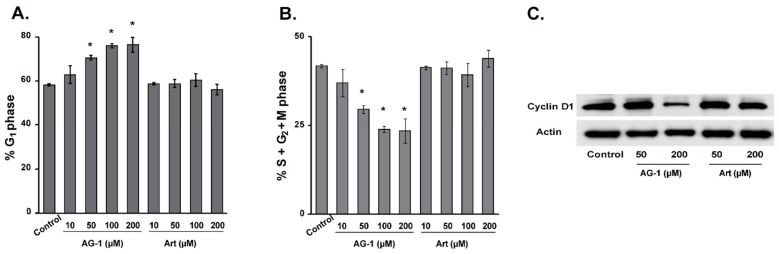
Artemisinin dimer (AG-1) treatment decreases cyclin D1 protein expression resulting in an increase in the percentage of cells in the G_1_-phase of the cell cycle. Exponentially growing asynchronous cultures of MB-231 cells were treated for 72 hours with 10–200 µM of Art or AG-1. Ethanol-fixed cells were treated with RNase A and propidium iodide (PI). Flow cytometry was used to measure DNA content of cells. The percentage of cells in the (**A**) G_1_-phase and (**B**) S + G_2_ + M phases was calculated using MODFIT software. Results are presented as average and standard deviation. Asterisks represent significance compared to untreated control cells; *n* = 3, *p* < 0.05. (**C**) Western blot analysis of cyclin D1 protein expression in Art- and AG-1-treated MB231 cells.

To determine whether the growth-inhibitory property of AG-1 in cancer cells (Figure 2 and Figure 3) is due to its ability to perturb progression through the cell cycle, a flow cytometry assay was used to measure the percentage of cells in different phases of the cell cycle. MB-231 cells were treated with Art and AG-1 (0–200 µM) for 72 h, trypsinized and fixed in ethanol. Cells were incubated with RNaseA and propidium iodide (PI) prior to analysis of DNA content [11,17]. Results showed no significant change in cell cycle progression in in Art-treated cells compared to untreated control cells (Figure 4). However, treatment with AG-1 resulted in a dose-dependent increase in the percentage of G_1_-cells compared to untreated control (Figure 4A): approximately 60% in control vs. more than 75% in 200 µM AG-1-treated cells. The increase in the percentage of cells in the G_1_-phase was accompanied with a corresponding decrease in the percentage of cells in the S + G_2_ + M phases (Figure 4B). It is interesting to note that the AG-1-induced G_1_-accumulation was accompanied with a significant decrease in cyclin D1 protein levels; approximately 5 folds decrease in 200 µM AG-1-treated cells compared to cyclin D1 protein levels in Art treated and control cells (Figure 4C). These results suggest that the growth-inhibitory property of AG-1 could be due to its ability to decrease cyclin D1 protein expression that results in a significant increase in the percentage of G_1_-cells causing a delay in progression.

**Figure 5:**
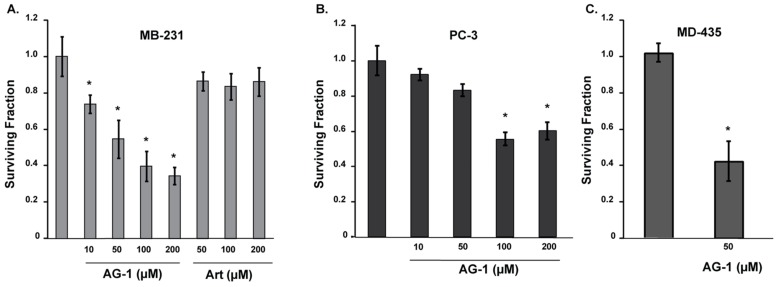
Artemisinin dimer (AG-1) treatment is more cytotoxic than Art. Asynchronous cultures of human (**A**) MB-231 breast cancer, (**B**) PC-3 prostate cancer and (**C**) MD-435 melanoma cancer cells were treated for 72 hours with Art or AG-1. Monolayer cultures were trypsinized and re-plated at limiting dilutions designed to plate single cells. Cultures were continued for 14 days in absence of the drugs. Cells were fixed and stained with crystal violet. Surviving fraction was calculated after correction for plating efficiency. Results are presented as average and standard deviation. Asterisks represent significance compared to untreated control cells; *n* = 3, *p* < 0.05.

The inhibition of cell proliferation in AG-1-treated cells could be due to a combination of cytostatic (Figure 2 and Figure 3) and cytotoxic effects. To determine whether AG-1 treatment is cytotoxic to cancer cells, a clonogenic assay was used to measure survival of single cells. Monolayer cultures of control and drug-treated cells were trypsinized and re-plated at limited dilutions designed to plate single cells. Cells were continued in culture for 14 d using regular medium without Art or AG-1. Surviving fraction (SF) was calculated after correction for plating efficiency (Figure 5). Results showed that treatment of cells with Art had minimal effect on SF, approximately 20% decrease. This decrease in SF did not change when the concentration of Art was increased to 100 and 200 µM. In contrast to Art, treatment with AG-1 showed a dose-dependent decrease in SF: approximately 25% in 10 µM, 50% in 50 µM, 65% in 100 µM, and 75% in 200 µM AG-1-treated cells (Figure 5A). Comparable results were also obtained when these experiments were performed using two separate cancer cell lines, PC-3 human prostate cancer cells and MD-435 human melanoma cells (Figure 5B,C). Taken together, results from Figure 2, Figure 3, Figure 4 and Figure 5 demonstrate that AG-1 is more potent at inhibiting proliferation and inducing toxicity of human cancer cells, and that perturbations in cell cycle progression may have a role in this process. 

### 3.3. Reactive Oxygen Species (ROS: Superoxide and Hydrogen Peroxide) Regulate AG-1-induced Toxicity

**Figure 6:**
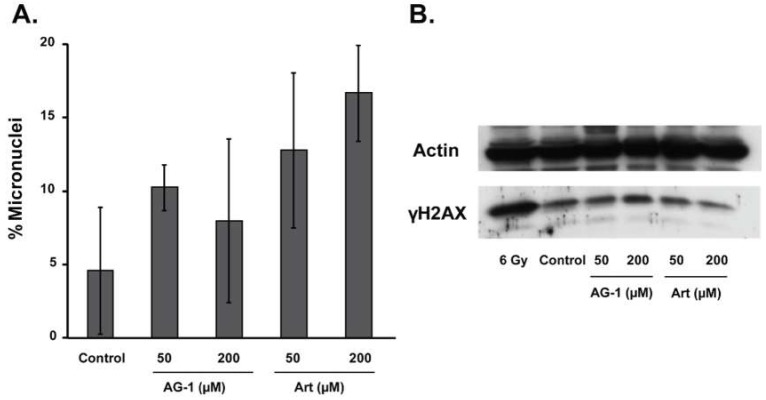
Artemisinin dimer (AG-1)-induced cytotoxicity appears to be independent of DNA damage measured at 72 hours post-treatment. (**A**) AG-1 and Art treatments did not cause any significant change in the percentage of micronuclei. Asynchronous cultures of MB-231 cells were treated for 72 hours with 50–200 µM of Art or AG-1 and then incubated with cytochalasin-B followed by staining with acridine orange. Micronuclei in binucleated cells with well-preserved cytoplasm were scored using microscopy. (**B**) AG-1 and Art treatments did not cause any significant change in the levels of phosphorylated H2AX (γH2AX). Total cellular protein extracts isolated from 72 hours control, Art- and AG-1-treated cells were separated by SDS-PAGE and transferred onto a nitrocellulose membrane. The membrane was then incubated with antibodies to γH2AX and actin.

To better understand the mechanisms regulating AG-1-mediated cytotoxic effects, we first investigated DNA damage endpoints by measuring the frequency of micronuclei and phosphorylated-H2AX (γH2AX) protein levels—two of the most commonly used markers of DNA damage. For the micronuclei assay, cells were treated for 72 h with Art or AG-1 and then blocked at mitosis by incubating cells with cytochalasin-B. Cells were stained with acridine orange and the number of micronuclei per binucleated cell was counted using microscopy [15,18]. Results showed no statistically significant difference in the percentage of micronuclei in Art- and AG-1-treated cells compared to untreated control at 72 h post-treatment (Figure 6A). Consistent with this observation, results from western-blot analysis also showed no significant difference in the protein levels of γH2AX in Art- and AG-1-treated cells compared to untreated control (Figure 6B). The increase in the protein levels of γH2AX in 6-Gy irradiated cells was used as a positive control for DNA damage. These results suggest that the cytotoxic effects of AG-1 may be independent of the DNA damage measured at 72 h post-treatment.

**Figure 7:**
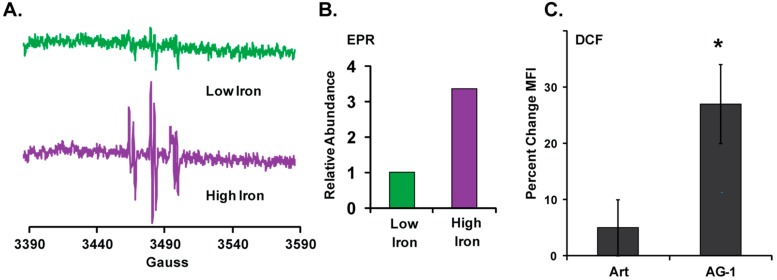
Artemisinin dimer (AG-1) generates carbon-centered radicals in cell-free system, and it increases the steady-state levels of cellular reactive oxygen species (ROS). (**A**) Detection of AG-1 derived carbon-centered radicals in cell-free system. AG-1 (1 mM) was incubated in phosphate buffer saline (pH 7.4) containing 25 mM POBN (α-(4-pyridyl-1-oxide)-*N*-*tert*-butylnitrone) in the presence of 20 µM or 100 µM ferrous sulfate. EPR (Electron Paramagnetic Resonance) spectra have the characteristics of spin adducts of two different carbon-centered radicals (radical-1, a^N^ = 16.0 G, a^H^ = 2.8 G; radical-2, a^N^ = 14.7 G, a^H^ = 2.4 G with a g-shift of about 0.25 G and a concentration ratio close to 1:1). Representative spectra showing AG-1 derived carbon-centered radicals are shown. (**B**) Bar graph represents relative abundance of carbon-centered radicals in the presence of low (20 µM) and high (100 µM) concentrations of ferrous sulfate. (**C**) AG-1 treatment increases the intracellular flux of oxidants. MB-231 cells were treated with 50 µM of Art or AG-1 for 15 minutes and then incubated with H_2_DCF-DA (5-(and-6)-carboxy-2′,7′-dichlorodihydrofluorescein diacetate). H_2_DCF-oxidation, i.e., formation of DCF, was measured by flow cytometry and percent change in MFI (Mean Fluorescence Intensity) was calculated relative to untreated controls for individual treatments. Results are presented as average and standard deviation. Asterisks represent significance compared to Art-treated cells; *n* = 3, *p* < 0.05.

In the presence of iron, the endoperoxide (–C–O–O–C–)-bridge in Art and AG-1 is believed to undergo a one-electron reductive cleavage to generate carbon- and oxygen-centered radicals, which in a cellular context can lead to oxidative stress. To determine whether AG-1 in the presence of Fe^2+^ can generate carbon-centered radicals, electron paramagnetic resonance (EPR) spectroscopy was used to detect the formation of AG-1 carbon-centered radical in a cell-free system. AG-1 (1 mM) was incubated in PBS (pH 7.4) containing 25 mM POBN in the presence of 20 µM or 100 µM ferrous sulfate. EPR spectra have the characteristics of spin adducts of two different carbon-centered radical, as captured by POBN (radical-1, a^N^ = 16.0 G, a^H^ = 2.8 G; radical-2, a^N^ = 14.7 G, a^H^ = 2.4 G) with a g-shift of about 0.25 G and a concentration ratio close to 1:1 (Figure 7A). The hyperfine splitting parameters of these two spin-adducts of POBN are consistent with the spin trapping of carbon centered radicals [13,19]. The relative abundance of the AG-1 carbon-centered radicals increased approximately 3-fold in the presence of 100 µM (high) compared to 20 µM (low) ferrous sulfate (Figure 7B), consistent with the one-electron reductive cleavage of an endoperoxide.

In the presence of molecular oxygen, the carbon-centered radicals of AG-1 can add dioxygen to form peroxyl radicals. These could in turn lead to the formation of superoxide, which can be converted to hydrogen peroxide by superoxide dismutase [20]. Hydrogen peroxide can be sensed using a flow cytometry assay designed to measure oxidation of a redox-sensitive reporter compound such as H_2_DCF-DA. H_2_DCF-DA is routinely used to probe for intracellular redox status (e.g., hydrogen peroxide). H_2_DCF-DA is a non-fluorescent molecule that passively enters cells. Following uptake, intracellular esterases remove the acetate groups in H_2_DCF-DA, converting it to H_2_DCF; this charged species is trapped in cells and can be oxidized by cellular oxidants to a green-fluorescent product (DCF) that can be quantitated using flow cytometry. The mean fluorescence intensity (MFI) of twenty thousand cells was used to assess intracellular redox status. To determine whether Art and AG-1 treatment perturbs intracellular redox status, cells were treated with 50 µM of Art or AG-1 for 15 min followed by incubation with 20 µM H_2_DCF-DA. DCF formation was measured by flow cytometry and percent change in MFI calculated relative to untreated control (Figure 7C). Results showed that treatment with Art minimally perturbed the intercellular redox status (<10% increase in MFI vs. control). However, treatment with AG-1 significantly shifted cellular redox status towards a more oxidized environment (≈30% increase in MFI). These results indicate that treatment with AG-1 induces greater oxidative stress than treatment with Art.

**Figure 8:**
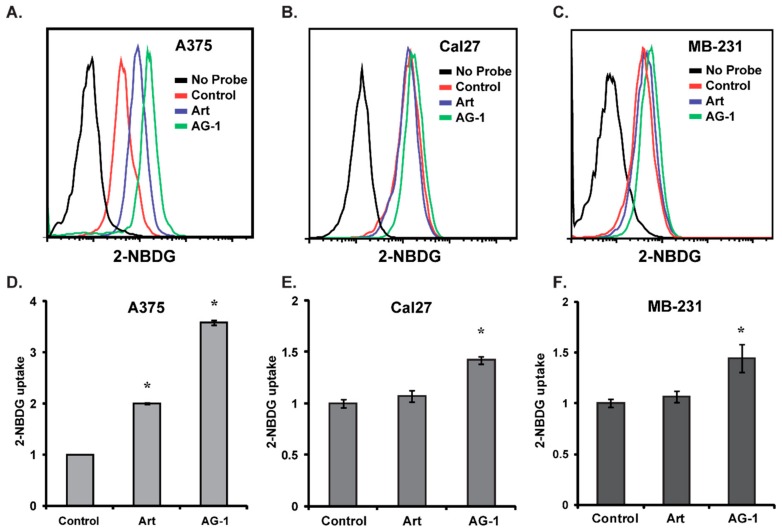
Higher glucose uptake in artemisinin dimer (AG-1)-treated human cancer cells. Asynchronous cultures of (**A,D**) A375 melanoma, (**B,E**) Cal27 head–neck and (**C,F**) MB-231 breast cancer cells were treated with 100 µM Art or AG-1 for 24 hours. Cells were rinsed and incubated with DMEM (Dulbecco’s Modified Eagles Media) containing 20 µM of 2-NBDG (2-(*N*-(7-nitrobenz-2-oxa-1,3-diazol-4-yl)amino)-2-deoxyglucose) for 1 hour. 2-NBDG fluorescence was measured using flow cytometry. (**A–C**) show representative flow cytometry histograms and quantitative data are shown in (**D–F**). Asterisks represent significance compared to untreated control cells; *n* = 3, *p* < 0.05.

To further determine the induction of oxidative stress in AG-1-treated cancer cells, we used a flow cytometry based assay to measure glucose uptake in control and drug-treated cells (Figure 8). We previously reported increases in glucose uptake and glucose consumption rates in oxidatively stressed cells [12,21]. Control and drug-treated asynchronous cultures of A375, Cal27 and MB-231 cells were incubated with 2-NBDG for 1 h. Flow cytometry was used to measure fluorescence of 2-NBDG, an indicator of glucose uptake. Results showed significant increases in 2-NBDG fluorescence in AG-1-treated cells compared to 2-NBDG uptake in control and Art treated cells (Figure 8A–F). In A375 cells, Art treatment also resulted in an increase in 2-NBDG fluorescence, albeit at a much lower level compared to treatment with AG-1 (Figure 8D). Overall, these results (Figure 7 and Figure 8) show that the AG-1 treatments shift cancer cell redox status more towards an oxidizing environment, which also correlated with increases in AG-1-induced toxicity of cancer cells (Figure 5).

**Figure 9:**
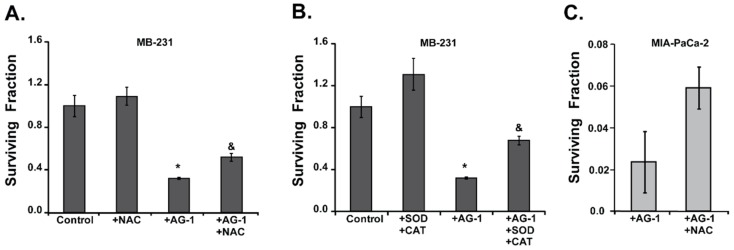
Oxidative stress regulates artemisinin dimer (AG-1)-induced cytotoxicity. (**A**) Pre-treatment with a thiol-antioxidant suppresses AG-1-induced cytotoxicity. Asynchronous cultures of MB-231 cells were pre-treated for 3 hours with 5 mM NAC (N-Acetyl-L-Cysteine) and then treated for 72 hours with 200 µM of AG-1. (**B**) Antioxidant enzymes inhibit AG-1-induced cytotoxicity. Asynchronous cultures of MB-231 cells were pre-treated for 3 hours with polyethylene glycol conjugated catalase (500 units mL^−1^) and superoxide dismutase (50 units mL^−1^) and then incubated with 200 µM of AG-1. (**C**) MIA PaCa-2 cells were treated with 5 mM NAC 3 hours prior to treatment with 5 µM AG-1. A clonogenic assay was used to measure cytotoxicity. Asterisks represent significance compared to untreated control cells; *n* = 3, *p* < 0.05. Ampersand (&) represents significance compared to cells that were treated with AG-1.

The causality of oxidative stress resulting in AG-1-induced toxicity of cancer cells is clearly evident from the results shown in Figure 9. Cells were pre-treated with a small molecular weight thiol-antioxidant, *N*-acetyl-l-cysteine (NAC, 5 mM) for 3 h followed by treatment with 200 µM of AG-1 for 72 h. NAC is a widely used thiol-antioxidant that can enhance the ability of cells to scavenge hydroperoxides. Toxicity was measured using a clonogenic assay. As shown above (Figure 5), AG-1 treatment significantly reduced (approximately 70%) cell survival compared to survival of untreated control cells (Figure 9A). 

It is interesting to note that pre-treatment with NAC significantly suppressed AG-1-induced toxicity in MB-231 and MIA PaCa-2 cells (Figure 9A,C). Comparable results (Figure 9B) were also obtained in cells pre-treated with antioxidant enzymes that are well-known to remove superoxide (superoxide dismutase) and hydrogen peroxide (catalase). Cells were pre-treated with PEG-superoxide dismutase (SOD) and PEG-catalase (CAT) followed by incubation with 200 µM of AG-1 for 72 h. Cell survival was measured using a clonogenic assay. Pre-treatment with SOD and CAT significantly suppressed AG-1-induced toxicity: approximately 70% decrease in cell survival in AG-1-treated cells compared to 50% decrease in survival in AG-1-treated cells that were pre-treated with SOD and CAT (Figure 9B). These results demonstrate that treatment with AG-1 induces oxidative stress presumably due to the generation of carbon- and oxygen-centered radicals resulting in an increase in cellular ROS levels. Suppression of AG-1-induced toxicity in cancer cells pretreated with antioxidants suggests that ROS (superoxide and hydrogen peroxide) mediate the anti-proliferative effects of AG-1. 

## 4. Discussion

In this study, we synthesized the novel C-16 carba-dimer (AG-1) from artemisitene by using a nitroaliphatic chemistry approach. The synthesized AG-1 was primarily C-10 acetal or C-10 carba-dimers. We believe that the introduction of a nitro chromophore into the artemisinin dimer is unique to our synthesis approach. Results from in vitro cell culture experiments showed that AG-1 is more cytotoxic than Art to human cancer cells. The toxicity of AG-1 appears to be mediated by oxidative stress presumably due to the generation of AG-1 carbon- and oxygen-centered radicals, which results in an increase in cellular ROS levels (superoxide and hydrogen peroxide). Suppression of AG-1-induced toxicity with antioxidants that scavenge ROS suggests that superoxide and hydrogen peroxide mediate the toxicity of AG-1 in cancer cells.

**Figure 10:**
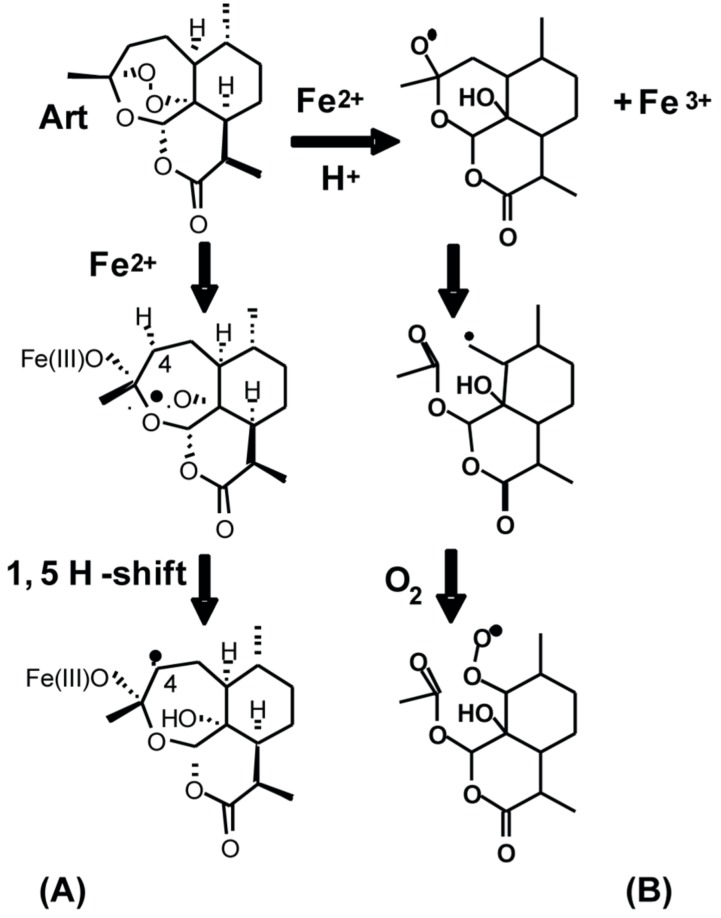
Pathways of artemisinin (**A**) carbon and (**B**) oxygen centered radical formation in cell-free system.

In recent years, there has been a growing interest in the application of anti-malarial drugs (artemisinin and its derivatives) as anti-cancer agents because their mode of action is believed to be due to the iron-mediated formation of carbon- and oxygen-centered radicals [6,22,23,24], which can lead to the generation of ROS (e.g., superoxide, hydrogen peroxide, peroxyl radicals, etc.) resulting in oxidative stress. Fe^2+^-mediated cleavage of the endoperoxide-bridge in Art and AG-1 can result in the generation of an oxygen-centered radical, which undergoes 1,5 hydrogen shift [2,10] to form a more stable carbon-centered radical (Figure 10A). Indeed, results from EPR spectroscopy showed the formation of AG-1 derived carbon-centered radicals in a cell-free system that is supplemented with iron (Figure 7A). The dose-dependent increase in the amount of the AG-1 carbon-centered radicals further supports the role of iron in this reaction (Figure 7B).

An additional pathway of free radical formation in the presence of dioxygen could be due to the Fe^2+^-mediated generation of superoxide (or peroxyl radical) and an epoxide of artemisinin (Figure 10B). Both superoxide and epoxide are anticipated to cause oxidative stress resulting in damage to cellular macromolecules and, subsequently, cell death. Cancer cells have a higher demand for iron to meet their biochemical activities essential for proliferation [25]. Iron is required for proper functioning of many of the proteins regulating proliferation (e.g., R2-subunit of ribonucleotide reductase) and cellular metabolism (e.g., Fe–S clusters of the electron transport chain, aconitase, and prolyl-hydroxylases). Iron chelators are known to suppress cyclin D1 accumulation and inhibit ribonucleotide reductase activity leading to a G_1_-arrest [26,27]. Under physiological conditions iron homeostasis is a tightly regulated process of uptake, utilization, and storage that are orchestrated by the transferrin receptor, divalent metal transporter 1, and ferritin proteins [28,29,30]. Furthermore, there is a weakly chelated form of iron (labile iron pool, also known as chelatable iron) that may be involved in intracellular trafficking [29]. The labile iron pool can also result from superoxide-mediated release of iron from certain proteins, e.g., Fe_4_–S_4_ cluster in aconitase [31]. Iron-driven one-electron reductions of oxygen leads to the formation of hydroxyl radical (Fenton/Haber–Weiss reaction), which can damage cellular macromolecules. Therefore, it is anticipated that in a cellular context, iron-catalyzed formation of AG-1-oxygen-centered radicals may lead to an increase in cellular ROS levels resulting in oxidative stress and toxicity. Indeed, cellular ROS levels, determined by flow cytometry measurements of the oxidation of H_2_DCF to DCF, were found to be significantly higher in AG-1-treated MB-231 cells compared to Art-treated cells (Figure 7C). These results suggest that AG-1 treatments increased intracellular ROS levels more than Art-treated cells.

Intracellular ROS levels are intimately related to cellular processes including proliferation, differentiation, and cell death [32]. ROS (superoxide and hydrogen peroxide) are oxygen containing molecules that are highly reactive in oxidation and reduction (redox) reactions. Superoxide (O_2_•^−^) can be converted to hydrogen peroxide (H_2_O_2_) spontaneously as well as by superoxide dismutase (SOD) antioxidant enzymes. Mammalian cells have three forms of SOD: MnSOD in mitochondria; CuZnSOD in cytoplasm and nucleus; and EcSOD in plasma membrane [33,34]. Hydrogen peroxide is converted to water by catalase (CAT) and glutathione peroxide (GPx) family of enzymes [35,36]. Superoxide and H_2_O_2_ react with transition metal ions (e.g., cuprous and ferrous ions) through Fenton and Haber–Weiss chemistry generating the highly reactive hydroxyl radical (HO•) that can damage cellular macromolecules resulting in cell death [37]. 

**Figure 11:**
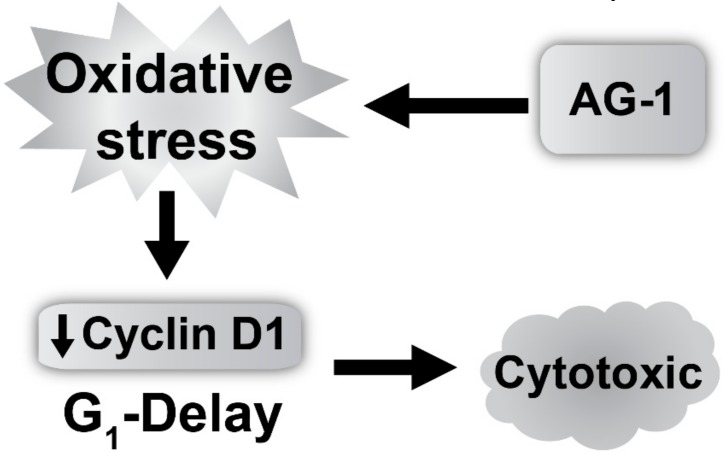
An illustration of AG-1 inducing oxidative stress and G_1_ delay resulting in toxicity.

ROS were traditionally thought of as toxic byproducts of living in an aerobic environment. However, recent evidence also suggests that ROS act as second messengers regulating numerous signaling pathways that regulate cellular processes including proliferation and cell death [32,38]. For example, NIH 3T3 murine fibroblasts treated with 0.02–0.13 μM H_2_O_2_ enhanced proliferation while treatment with 0.25–2 μM H_2_O_2_ resulted in cell death [39]. Similarly, a prooxidant event in the G_1_-phase is required for cells to enter S-phase; suppression of this prooxidant event inhibited cells entry into S-phase [40,41]. This dual function of ROS, proliferation and cell death, could result from the difference in their concentration, pulse duration, and sub-cellular localization. ROS levels below a threshold are pro-proliferative, while ROS levels above this threshold are cytostatic and cytotoxic. Consistent with this premise, results show that AG-1-induced increase in cellular ROS levels (Figure 7) was associated with a significant decrease in proliferation (Figure 2 and Figure 3) and increase in cell death (Figure 5 and Figure 9). The decrease in proliferation was associated with a significant increase in the percentage of cells in the G_1_-phase and decrease in cyclin D1 protein expression (Figure 4). Interestingly, AG-1-induced cytostatic and cytotoxic effects appear to be independent of DNA damage at 72 h post-treatment (Figure 6). However, pre-treatment of cancer cells with antioxidants, NAC and antioxidant enzymes significantly suppressed AG-1-induced toxicity (Figure 9). These results suggest that ROS (O_2_•^−^ and H_2_O_2_) mediate AG-1-induced toxicity in cancer cells (Figure 11).

## 5. Conclusions

The present study used a selenoxide elimination method to synthesize an artemisinin-derived carba-dimer (AG-1) and determined its anti-proliferative effects in human normal and cancer cells. Results showed that compared to Art, AG-1 is more cytotoxic to cancer cells. Both compounds were non-toxic to normal human fibroblasts. The toxicity of AG-1 in cancer cells appears to be due to its ability to shift cellular redox status to a more oxidized environment that is accompanied with a significant decrease in cyclin D1 protein expression and increase in the percentage of cells in the G_1_-phase of the cell cycle.
